# WHO method for estimating congenital syphilis to inform surveillance and service provision, Paraguay

**DOI:** 10.2471/BLT.20.271569

**Published:** 2022-01-25

**Authors:** Katherine Heath, Monica Alonso, Gloria Aguilar, Tania Samudio, Eline Korenromp, Jane Rowley, Anita Suleiman, Ye Yu Shwe, Khin Cho Win Htin, Naoko Ishikawa, Morkor Newman Owiredu, Melanie Taylor

**Affiliations:** a Burnet Institute, 85 Commercial Road, Melbourne, Victoria, 3004, Australia.; b World Health Organization Regional Office for the Americas, Washington, DC, United States of America (USA).; c National Human Immunodeficiency Virus (HIV) and Sexually Transmitted Infection (STI) Program, Ministry of Health, Asunción, Paraguay.; d Avenir Health, Geneva, Switzerland.; e Department of Global HIV, Hepatitis and Sexually Transmitted Infections Programmes, World Health Organization, Geneva, Switzerland.; f National HIV/STI Program, Ministry of Health, Putrajaya, Malaysia.; g Asia and the Pacific Regional Office, Joint United Nations Programme on HIV/AIDS (UNAIDS), Bangkok, Thailand.; h UNAIDS Country Office, Phnom Penh, Cambodia.; i WHO Regional Office for the Western Pacific, Manila, Philippines.; j Department of Sexually Transmitted Disease Prevention, United States Centers for Disease Control and Prevention, Atlanta, Georgia, USA.

## Abstract

**Problem:**

In Paraguay, incomplete surveillance data resulted in the burden of congenital syphilis being underestimated, which, in turn, led to missed opportunities for infant diagnosis and treatment.

**Approach:**

The prevalence of congenital syphilis, as defined by the World Health Organization (WHO), was estimated for Paraguay using the WHO congenital syphilis estimation tool. This tool was also used to monitor progress towards the elimination of mother-to-child transmission of syphilis.

**Local setting:**

The burden of syphilis in Paraguay has historically been high: its prevalence in pregnant women was estimated to be 3% in 2018.

**Relevant changes:**

The incidence rate of congenital syphilis estimated using the WHO tool was around nine times the reported prevalence. Subsequently, Paraguay: (i) provided training to improve diagnosis and case reporting; (ii) strengthened information systems for case monitoring and reporting; and (iii) procured additional rapid dual HIV–syphilis and rapid plasma reagin tests to increase syphilis testing capacity. In addition, the Ministry of Health prepared a new national plan for eliminating mother-to-child transmission of syphilis, with clear monitoring milestones.

**Lessons learnt:**

Health-care providers’ reporting and surveillance procedures for congenital syphilis may not adequately reflect national and international case definitions. Use of the WHO congenital syphilis estimation tool in Paraguay drew attention to congenital syphilis as a national public health problem and highlighted the importance of comprehensive national surveillance systems and accurate data. Ongoing use of the WHO tool can track progress towards the elimination of mother-to-child transmission of syphilis by helping improve syphilis service coverage and national surveillance.

## Résumé

**Problème:**

Au Paraguay, l'insuffisance de données de surveillance entraîne une sous-estimation du fardeau causé par la syphilis congénitale, ce qui aboutit à des occasions manquées de diagnostiquer et traiter les nourrissons.

**Approche:**

Le taux d'incidence de la syphilis congénitale, telle que définie par l'Organisation mondiale de la Santé (OMS), a été calculée au Paraguay à l'aide de l'outil d'évaluation de l'OMS. Cet outil a également servi à suivre les progrès dans l'élimination de la transmission de la syphilis de la mère à l'enfant.

**Environnement local:**

Le fardeau de la syphilis au Paraguay a toujours été lourd: la prévalence de la maladie chez les femmes enceintes était estimée à 3% en 2018.

**Changements significatifs:**

Le taux de prévalence de la syphilis congénitale calculé avec l'outil de l'OMS était environ neuf fois plus élevé que celui enregistré. Par conséquent, le Paraguay a: (i) proposé des formations afin d'améliorer le diagnostic et le signalement des cas; (ii) renforcé les systèmes d'information pour le suivi et la notification des cas; et enfin, (iii) fourni des doubles tests rapides VIH/syphilis et des tests rapides de la réagine plasmatique supplémentaires en vue d'accroître les capacités de dépistage de la syphilis. En outre, le ministère de la Santé a élaboré un nouveau plan national destiné à éliminer la transmission de la syphilis de la mère à l'enfant, comprenant une série d'étapes de suivi.

**Leçons tirées:**

Les procédures des prestataires de soins de santé en matière de notification et de surveillance de la syphilis congénitale pourraient différer de la définition des cas aux niveaux national et international. L'utilisation de l'outil d'évaluation mis au point par l'OMS pour la syphilis congénitale au Paraguay a attiré l'attention sur cette maladie qui représente un problème de santé publique nationale, et souligné l'importance des systèmes de surveillance complets et des données précises. Continuer à employer cet outil peut permettre de mesurer les progrès accomplis dans l'élimination de la transmission de la syphilis de la mère à l'enfant, en contribuant à améliorer la couverture des services et la surveillance nationale.

## Resumen

**Situación:**

En Paraguay, los datos de vigilancia incompletos ocasionaron que se subestimara la carga de sífilis congénita, lo que, a su vez, hizo que se perdieran las oportunidades de diagnóstico y tratamiento de los lactantes.

**Enfoque:**

La prevalencia de la sífilis congénita, según la definición de la Organización Mundial de la Salud (OMS), se estimó para Paraguay utilizando la herramienta de estimación de la sífilis congénita de la OMS. Esta herramienta también se utilizó para monitorear el progreso hacia la eliminación de la transmisión maternofilial de la sífilis.

**Marco regional:**

La carga de sífilis en Paraguay ha sido históricamente alta: su prevalencia en embarazadas se estimó en un 3 % en 2018.

**Cambios importantes:**

La tasa de incidencia de sífilis congénita estimada con la herramienta de la OMS era aproximadamente nueve veces superior a la notificada. En consecuencia, Paraguay: i) impartió formación para mejorar el diagnóstico y la notificación de los casos; ii) reforzó los sistemas de información para el monitoreo y la notificación de los casos; y iii) adquirió más pruebas rápidas duales de VIH-sífilis y de inmunoglobulina E plasmática rápida para aumentar la capacidad de realizar pruebas de sífilis. Además, el Ministerio de Salud Pública preparó un nuevo plan nacional para eliminar la transmisión maternofilial de la sífilis, con claros hitos de monitoreo.

**Lecciones aprendidas:**

Es posible que los procedimientos de notificación y vigilancia de la sífilis congénita que aplican los profesionales sanitarios no reflejen de manera adecuada las definiciones nacionales e internacionales de los casos. El uso de la herramienta de estimación de la sífilis congénita de la OMS en Paraguay llamó la atención sobre la sífilis congénita como un problema nacional de salud pública y destacó la importancia de contar con sistemas nacionales de vigilancia integrales y datos precisos. El uso constante de la herramienta de la OMS puede hacer un seguimiento de los avances hacia la eliminación de la transmisión maternofilial de la sífilis al ayudar a mejorar la cobertura de los servicios relacionados con la sífilis y la vigilancia nacional.

## ملخص

**المشكلة:**

أدت بيانات المراقبة غير المكتملة في باراغواي إلى الحد من عبء تقييم الزهري الخلقي، مما أدى بدوره إلى ضياع فرص تشخيص الرضع وعلاجهم.

**الأسلوب:**

تم تقييم انتشار مرض الزهري الخلقي، وفقًا لتعريف منظمة الصحة العالمية (WHO)، في باراغواي باستخدام أداة تقييم الزهري الخلقي التابعة لمنظمة الصحة العالمية. تم استخدام هذه الأداة أيضًا لرصد التقدم نحو القضاء على انتقال مرض الزهري من الأم إلى الطفل.

**المواقع المحلية:**

كان عبء مرض الزهري في باراغواي مرتفعًا على مدى تاريخها، فقد تم تقدير انتشاره بين النساء الحوامل بنسبة 3% في عام 2018.

**التغيّرات:**

**ذات** **الصلة ** كان معدل انتشار الزهري الخلقي، الذي تم تقييمه باستخدام أداة منظمة الصحة العالمية، حوالي تسعة أضعاف الانتشار المُبلغ عنه. وبالتالي فقد قامت باراغواي بما يلي: (1) توفير التدريب لتحسين التشخيص والإبلاغ عن الحالات؛ و(2) تعزيز نظم المعلومات لرصد الحالات والإبلاغ عنها؛ و(3) شراء اختبارات إضافية سريعة مزدوجة لفيروس نقص المناعة البشرية والزهري، واختبارات البلازما السريعة لزيادة القدرة على اختبار الزهري. وبالإضافة إلى ذلك، فقد أعدت وزارة الصحة خطة وطنية جديدة للقضاء على انتقال مرض الزهري من الأم إلى الطفل، مع مراحل مراقبة واضحة.

**الدروس:**

**المستفادة ** قد لا تعكس إجراءات الإبلاغ والمراقبة لدى مقدمي الرعاية الصحية، والخاصة بمرض الزهري الخلقي، تعريفات الحالة الوطنية والدولية بشكل كافٍ. إن استخدام أداة تقييم الزهري الخلقي التابعة لمنظمة الصحة العالمية في باراغواي، قد لفت الانتباه إلى الزهري الخلقي باعتباره مشكلة وطنية للصحة العامة، وأكّد على أهمية أنظمة المراقبة الوطنية الشاملة والبيانات الدقيقة. إن الاستخدام المستمر لأداة منظمة الصحة العالمية يمكنه أن يتتبع التقدم نحو القضاء على انتقال مرض الزهري من الأم إلى الطفل، وذلك من خلال المساعدة في تحسين تغطية خدمة مرض الزهري والمراقبة الوطنية.

## 摘要

**问题:**

在巴拉圭，不完整的监测数据导致先天性梅毒的负担被低估，由此导致错过婴儿诊断和治疗的机会。

**方法:**

根据世界卫生组织的 (WHO) 定义，采用世界卫生组织先天性梅毒评估工具来估算先天性梅毒的患病率。该工具还可用来监测梅毒母婴传播评估领域的进展情况。

**当地状况:**

巴拉圭的梅毒负担历来很高：2018 年的孕妇患病率估计占到 3%。

**相关变化:**

使用世界卫生组织工具评估的先天性梅毒患病率大约是所报告的患病率的九倍。随后，巴拉圭：(I) 开展了培训以提升诊断和病例报告；(ii) 加强了病例监测和报告的信息系统，并且 (iii) 采购了额外的快速双重 HIV-梅毒检测和快速血浆反应素试验以增强梅毒检测能力。除此以外，卫生部还制定了一项新的全国性计划，通过明确的监测里程碑来评估梅毒的母婴传播。

**经验教训:**

医疗护理机构的先天性梅毒报告和监测程序可能无法充分反映全国性和国际性的病例定义。在巴拉圭采用世界卫生组织先天性梅毒评估工具提高了对先天性梅毒作为全国性公共卫生问题的关注度，强调了综合的全国性监测系统和准确数据的重要性。持续使用世界卫生组织工具可以有助于提高梅毒卫生服务覆盖面和全国性监测的普及，从而追赶梅毒母婴传播评估领域的进展。

## Резюме

**Проблема:**

В Парагвае неполные данные наблюдений привели к недооценке бремени врожденного сифилиса, что в свою очередь привело к упущенным возможностям в диагностике и лечении младенцев.

**Подход:**

В Парагвае распространенность врожденного сифилиса, по определению Всемирной организации здравоохранения (ВОЗ), оценивалась с использованием инструмента ВОЗ для оценки врожденного сифилиса. Этот инструмент также использовали для мониторинга прогресса в борьбе с передачей сифилиса от матери к ребенку.

**Местные условия:**

В Парагвае бремя сифилиса достигло рекордных показателей: его распространенность среди беременных женщин в 2018 году составила 3%.

**Осуществленные перемены:**

Распространенность врожденного сифилиса, оцененная с помощью инструмента ВОЗ, примерно в девять раз превышала зарегистрированную распространенность. В результате Парагвай: (i) организовал обучение для улучшения диагностики и отчетности о случаях заболевания; (ii) укрепил информационные системы для мониторинга случаев заболевания и отчетности; (iii) закупил дополнительные двойные экспресс-тесты на ВИЧ/сифилис и реакцию быстрого определения реагинов плазмы, чтобы расширить возможности тестирования на сифилис. Кроме того, Министерство здравоохранения подготовило новый национальный план по борьбе с передачей сифилиса от матери к ребенку с четкими этапами мониторинга.

**Выводы:**

Процедуры отчетности и наблюдения медицинских работников за врожденным сифилисом могут неадекватно отражать определения случаев заболевания на национальном и международном уровне. Использование инструмента ВОЗ для оценки врожденного сифилиса в Парагвае привлекло внимание к врожденному сифилису как национальной проблеме в сфере общественного здравоохранения и подчеркнуло важность комплексных национальных систем наблюдения и точных данных. Постоянное использование инструмента ВОЗ поможет отслеживать прогресс в борьбе с передачей сифилиса от матери к ребенку, способствуя большему охвату услугами по борьбе с сифилисом и наблюдению на национальном уровне.

## Introduction

Vertical transmission of syphilis results in adverse birth outcomes, including fetal death (stillbirth), neonatal death, preterm birth, a low birth weight and congenital infection.^1^ In Paraguay, the elimination of mother-to-child transmission of syphilis has been hampered by low coverage of maternal syphilis screening and treatment and by limited reporting of congenital syphilis.^2^ Historically, incomplete surveillance data have led to the burden of congenital syphilis being underestimated in the country and to missed opportunities for infant diagnosis and treatment.

In 2014 and 2017, the World Health Organization (WHO) released criteria to validate the elimination of mother-to-child transmission of human immunodeficiency virus (HIV) and syphilis.^3^^,^^4^ For syphilis, a country must have a congenital syphilis case rate of 50 or less per 100 000 live births and have reached the 95% service coverage targets outlined by WHO.^4^ By December 2021, 14 countries had received validation for the elimination of mother-to-child transmission of syphilis. The case rate target uses WHO’s surveillance case definition of congenital syphilis: (i) a live birth or fetal death at > 20 weeks of gestation or > 500 g (including stillbirth) born to a woman with positive syphilis serology and without adequate syphilis treatment; or (ii) a live birth, stillbirth or child aged < 2 years born to a woman with positive syphilis serology or with unknown serostatus, and with laboratory and/or radiographic and/or clinical evidence of syphilis infection (regardless of the timing or adequacy of maternal treatment).^4^

## Local setting

**Fig. 1:**
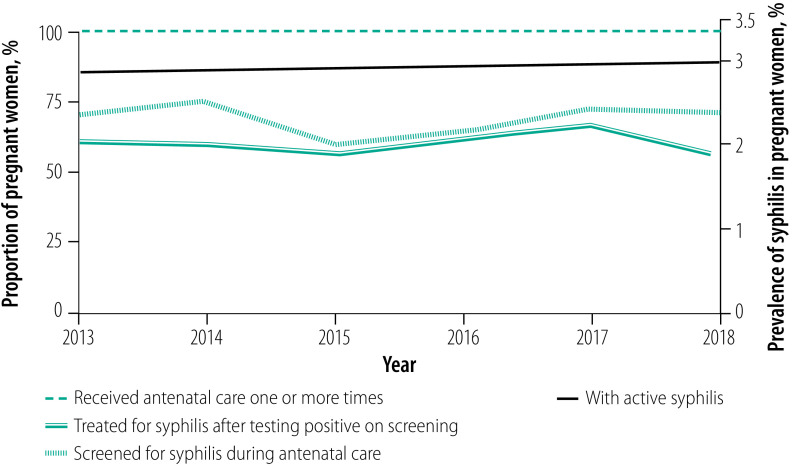
Coverage of antenatal services and syphilis prevalence in pregnant women, Paraguay, 2013–2018

Routine antenatal syphilis screening data from Paraguay indicated that the disease prevalence in pregnant women was 2.9% in 2016. The rate in 2018 was estimated to be 3.0% using a statistical prevalence time-trend model that combined all available antenatal syphilis surveillance data (i.e. from 2008 to 2016) with data from a sentinel survey in 2013.^5^ Country-reported indicators of the elimination of mother-to-child transmission in 2018 included: (i) 100.0% of pregnant women attended an antenatal care clinic at least once; (ii) 71.2% of women who received antenatal care were screened for syphilis; and (iii) 56.2% of pregnant women diagnosed with syphilis on screening were treated (Box 1 and Fig. 1).

Box 1 **Estimated congenital syphilis parameters, Paraguay, 2018**

- No. of live births: 141 896^a^
- Prevalence of active syphilis in pregnant women: 3.0%^b,c^
- Proportion of pregnant women who attended an antenatal care clinic at least once: 100.0%
- Proportion of pregnant women receiving antenatal care who were screened for syphilis: 71.2%
- Proportion of pregnant women diagnosed with syphilis during antenatal care who were adequately treated: 56.2%^d^
- Average gestational week of first syphilis treatment: 17^e^
- No. of congenital syphilis cases reported in national surveillance system: 280
- No. of congenital syphilis cases per 100 000 live births, derived using cases reported in the national surveillance system: 197
- No. of congenital syphilis cases estimated using the WHO congenital syphilis estimation tool: 2543
- No. of congenital syphilis cases per 100 000 live births derived from cases estimated using the WHO congenital syphilis estimation tool: 1792

WHO: World Health Organization.^a^ The number of live births in 2018 was used as a proxy measure for the number of pregnant women because data on live births were routinely estimated whereas data on the number of pregnant women were not.^b^ Active syphilis was defined as a positive result on both treponemal and nontreponemal tests. If only one test was used or the testing method was not known, a correction factor was applied as suggested by Ham et al.^6^^c^ The prevalence of maternal syphilis in Paraguay was estimated using the statistical trend-fitting model SPECTRUM STI (Avenir Health, Glastonbury, United States of America).^d^ Adequate treatment was defined as at least one dose of benzathine penicillin, 2.4 MU intramuscularly, at least 30 days before delivery.^e^ The national average gestational week of first attendance at an antenatal care clinic was used as a proxy measure for the time of testing and treatment.

## Approach

In 2016, WHO supported the development of a congenital syphilis estimation tool (hereafter referred to as the tool), which estimates the number of congenital syphilis cases in a country from data available on indicators of the elimination of mother-to-child transmission and additional data from users.^7^

In 2019, representatives from Paraguay attended a workshop organized by WHO and the Joint United Nations Programme on HIV/AIDS (UNAIDS) for countries in the WHO Region of the Americas, which included one day’s training on methods for estimating syphilis prevalence. During training, country representatives: (i) estimated the current prevalence of syphilis in their countries; (ii) reviewed national data on syphilis service coverage; (iii) learned to use the WHO tool; and (iv) employed the tool to estimate national trends in the incidence of congenital syphilis. One outcome of the workshop was a factsheet detailing the congenital syphilis burden in Paraguay.^5^

**Fig. 2:**
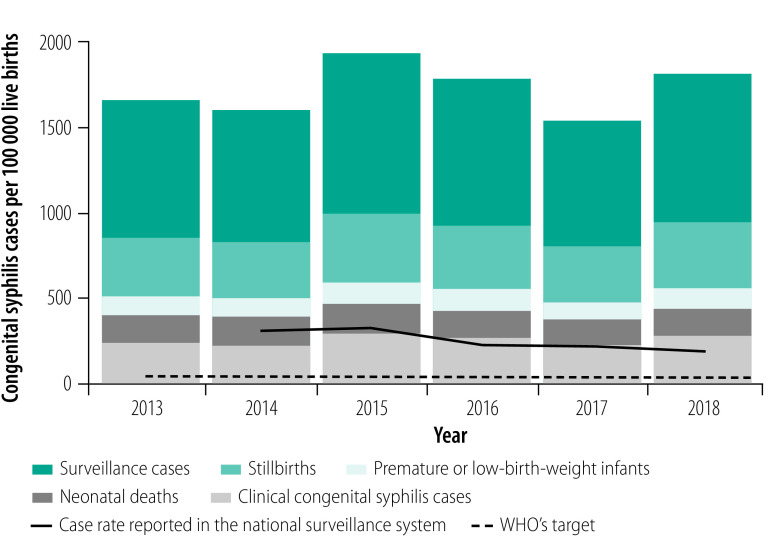
Estimated congenital syphilis case rate, Paraguay, 2013–2018

With the tool, the number of congenital syphilis cases in Paraguay in 2018 was estimated to be 2543 (1792 per 100 000 live births; Fig. 2). This number was substantially higher than the 280 cases (197 per 100 000 live births) reported for 2018. Both estimated and reported case numbers exceeded WHO’s threshold for the elimination of mother-to-child transmission, which is 50 cases per 100 000 live births. The approximately 9-fold discrepancy between estimated and reported cases suggested underdiagnosis and underreporting. Factors identified as contributing to this discrepancy included: (i) low syphilis screening and treatment coverage during antenatal care; (ii) the failure of surveillance systems to follow pregnant women with syphilis throughout both pregnancy and delivery, which resulted in infants not being recorded as having congenital syphilis; (iii) inadequate routine syphilis testing for mothers with adverse birth outcomes, which resulted in clinical cases being underreported; and (iv) restricted testing coverage in some areas due to limited access to laboratories. Therefore, although Paraguay’s case definition of congenital syphilis was similar to WHO’s definition, the existing case-reporting system did not capture all cases meeting the country’s definition.

WHO: World Health Organization.

Notes: The estimated congenital syphilis case rate (i.e. cases per 100 000 live births) in Paraguay was derived with the World Health Organization (WHO) congenital syphilis estimation tool, which uses data on maternal syphilis prevalence and on national service coverage of maternal syphilis screening and treatment. Congenital syphilis cases were disaggregated as: (i) adverse birth outcomes occurring in women with treated or untreated syphilis, which included stillbirths, premature or low-birth-weight infants, neonatal deaths and infants with clinical manifestations of congenital syphilis at birth (i.e. clinical congenital syphilis cases); and (ii) surveillance cases, which included all infants unaffected by an adverse birth outcome and without clinical manifestations of congenital syphilis at birth born to women with untreated syphilis. The reported case rate was obtained from national surveillance data on congenital syphilis. The threshold defined by WHO for the elimination of mother-to-child transmission of syphilis is 50 cases per 100 000 live births.

In 2020, after case numbers had been estimated using the tool, Paraguay modified its information systems to track mother–infant pairs. These systems now link data on pregnant women with syphilis to delivery outcomes, which reduces the risk that syphilis-exposed infants born to untreated mothers will not be recorded. According to WHO’s case definition, these infants are congenital syphilis cases. Previously they were underreported. As the changes were implemented by modifying existing systems, the costs incurred were minimal. An ongoing challenge will be sustained training of current and new staff to ensure the new systems are properly used. Between June and August 2021, training to improve the clinical diagnosis of congenital syphilis and case reporting was carried out at clinical sites in the five cities with the highest number of births and with large maternity hospitals: Asunción, Ciudad del Este, Coronel Oviedo, Encarnación and Pedro Juan Caballero. Over 600 clinical and statistics staff and staff involved in local and regional sexually transmitted infection programmes participated in 12 one-day workshops. As the workshops were added on to existing training, additional costs were minimal. Training covered the diagnosis and detection of congenital syphilis, in particular by: (i) reviewing mothers’ syphilis testing and treatment records; (ii) promoting the follow-up of mother–infant pairs; and (iii) syphilis testing of mothers experiencing abortions or stillbirths. It was emphasized that any infant born to a mother with untreated syphilis should be recorded as a congenital syphilis case.

In June 2021, the four maternity hospitals that see the highest numbers of pregnant women in the country were chosen as sentinel surveillance sites to monitor WHO’s indicators of the elimination of mother-to-child transmission of syphilis. Additional costs were minimal because existing local surveillance systems were used. In 2022, this surveillance system will be expanded to include more remote sentinel sites with indigenous populations by using integrated HIV resources from the Global Fund.

## Relevant changes

Due to improved surveillance and comprehensive training, the number of cases of congenital syphilis reported in Paraguay increased from 280 in 2018 to 541 in 2019 and 445 in 2020. These numbers are still below the 2543 cases estimated by the tool for 2018. However, additional planned changes will further improve case reporting and service coverage. Despite increased case reporting, syphilis screening and treatment coverage in 2019 was similar to coverage in 2018: (i) 70.0% of women who received antenatal care were screened for syphilis; and (ii) 58.0% of pregnant women diagnosed with syphilis on screening were treated.

In 2021, the Pan American Health Organization procured a large quantity of rapid dual HIV–syphilis test kits and rapid plasma reagin test kits for Paraguay. These kits will enable screening and confirmatory testing to be carried out in areas with limited access to laboratories and will facilitate testing during antenatal care visits outside of laboratory hours. In addition, the health ministry in Paraguay is implementing a new national plan to eliminate mother-to-child transmission of syphilis, with clear monitoring milestones and dedicated support.

Since 2019, Paraguay has been using the tool to monitor congenital syphilis cases and service coverage. Increased syphilis service coverage necessitates higher stocks of benzathine penicillin to treat newly diagnosed cases. Future use of the tool will: (i) support procurement planning by helping estimate the demand for benzathine penicillin; (ii) enable estimated and reported cases to be compared; and (iii) demonstrate the preventive effect of improved service coverage.

## Lessons learnt

In 2019, the WHO congenital syphilis estimation tool highlighted congenital syphilis as a public health problem in Paraguay (Box 2), thereby revitalizing political commitment to improved surveillance and service delivery.

Box 2 **Summary of main lessons learnt**

- Limited application of Paraguay’s case definition for congenital syphilis and partial misalignment with WHO’s case definition led to the underreporting of cases.
- Use of the WHO congenital syphilis estimation tool revealed a discrepancy between estimated and reported case numbers in Paraguay, thereby highlighting congenital syphilis as a national public health problem.
- Use of the WHO estimation tool resulted in: (i) the strengthening of Paraguay’s information systems; (ii) the provision of training to improve congenital syphilis diagnosis and case reporting; (iii) the procurement of additional rapid dual HIV–syphilis and rapid plasma reagin tests to increase testing capacity; and (iv) improvement in the monitoring of syphilis case rates and service coverage.

WHO: World Health Organization.

The tool’s estimates of the number of congenital syphilis cases were based on routinely collected, national data on: (i) the prevalence of maternal syphilis; (ii) antenatal care coverage; (iii) syphilis screening during antenatal care; and (iv) maternal syphilis treatment coverage. The quality of these data is known to vary widely between countries and regions. In Paraguay, use of the tool prompted improvements in data collection, data systems and national case reporting. In addition, it demonstrated that accurate and consistent diagnosis and case reporting and rigorous syphilis surveillance were critical components of national strategies for eliminating mother-to-child transmission.

Paraguay’s definition of a congenital syphilis case was similar to WHO’s definition but was not being applied to identify potential cases. Subsequently, case reporting was improved by training on syphilis detection and case management, which demonstrated that ensuring national surveillance systems align with WHO’s definition is a priority for tracking progress towards the elimination of mother-to-child transmission.

Scaling up rapid dual HIV–syphilis and rapid plasma reagin testing in Paraguay, in accordance with WHO’s recommendations,^8^ will result in higher detection rates and greater demand for benzathine penicillin. The ongoing incorporation of data on antenatal testing and treatment coverage and on maternal syphilis prevalence will enable the tool to provide estimates of the demand for benzathine penicillin, which is crucial for preventing medicine shortages.^9^

An ongoing challenge for high-prevalence countries is addressing syphilis transmission in general. Public health systems will have to prioritize syphilis prevention in both the general population and in high-risk groups alongside ensuring adequate clinical services. Reducing the prevalence of syphilis in the general population, including pregnant women, will have downstream effects for congenital syphilis.^10^

Other countries can use WHO’s freely available congenital syphilis estimation tool to demonstrate that high-quality data on antenatal care service coverage are important for syphilis surveillance and prevention programmes. In low-burden countries, such as Malaysia, Sri Lanka, the Maldives and Thailand, the tool has been used to validate the sustained elimination of mother-to-child transmission.^11^^–^^14^ Recently, WHO incorporated the tool into new guidance on eliminating mother-to-child transmission of syphilis and,^15^ by December 2021, WHO had provided technical assistance in use of the tool to at least 18 countries.
