# Corrigendum

**DOI:** 10.2471/BLT.22.110422

**Published:** 2022-04-01

In: Heath K, Alonso M, Aguilar G, Samudio T, Korenromp E, et al. WHO method for estimating congenital syphilis to inform surveillance and service provision, Paraguay. Bull World Health Organ. 2022 Mar 1; 100(3):231–36, 

On page 232, footnote to Fig. 1 should read as follows:

Note: Service coverage in pregnant women (left axis) is indicated by blue lines. Syphilis prevalence in pregnant women (right axis) is indicated by a black line.

