# Corrigendum

**DOI:** 10.2471/BLT.22.100522

**Published:** 2022-05-01

In: Heath K, Alonso M, Aguilar G, Samudio T, Korenromp E, et al. WHO method for estimating congenital syphilis to inform surveillance and service provision, Paraguay. Bull World Health Organ. 2022 Mar 1; 100(3):231–236, 

On pages 231, 234-236, the abstract and its translated versions should read as follows:

**WHO method for estimating congenital syphilis to inform surveillance and service provision, Paraguay**

**Abstract**

**Problem** In Paraguay, incomplete surveillance data resulted in the burden of congenital syphilis being underestimated, which, in turn, led to missed opportunities for infant diagnosis and treatment.

**Approach** The incidence of congenital syphilis, as defined by the World Health Organization (WHO), was estimated for Paraguay using the WHO congenital syphilis estimation tool. This tool was also used to monitor progress towards the elimination of mother-to-child transmission of syphilis.

**Local setting** The burden of syphilis in Paraguay has historically been high: its prevalence in pregnant women was estimated to be 3% in 2018.

**Relevant changes** The incidence rate of congenital syphilis estimated using the WHO tool was around nine times the reported incidence. Subsequently, Paraguay: (i) provided training to improve diagnosis and case reporting; (ii) strengthened information systems for case monitoring and reporting; and (iii) procured additional rapid dual HIV–syphilis and rapid plasma reagin tests to increase syphilis testing capacity. In addition, the Ministry of Health prepared a new national plan for eliminating mother-to-child transmission of syphilis, with clear monitoring milestones.

**Lessons learnt** Health-care providers’ reporting and surveillance procedures for congenital syphilis may not adequately reflect national and international case definitions. Use of the WHO congenital syphilis estimation tool in Paraguay drew attention to congenital syphilis as a national public health problem and highlighted the importance of comprehensive national surveillance systems and accurate data. Ongoing use of the WHO tool can track progress towards the elimination of mother-to-child transmission of syphilis by helping improve syphilis service coverage and national surveillance.

**Méthode de l'OMS pour mesurer la syphilis congénitale et orienter la surveillance et la prestation de services au Paraguay**

**Résumé**

**Problème** Au Paraguay, l'insuffisance de données de surveillance entraîne une sous-estimation du fardeau causé par la syphilis congénitale, ce qui aboutit à des occasions manquées de diagnostiquer et traiter les nourrissons.

**Approche** Le taux d'incidence de la syphilis congénitale, telle que définie par l'Organisation mondiale de la Santé (OMS), a été calculée au Paraguay à l'aide de l'outil d'évaluation de l'OMS. Cet outil a également servi à suivre les progrès dans l'élimination de la transmission de la syphilis de la mère à l'enfant.

**Environnement local** Le fardeau de la syphilis au Paraguay a toujours été lourd: la prévalence de la maladie chez les femmes enceintes était estimée à 3% en 2018.

**Changements significatifs** Le taux d'incidence de la syphilis congénitale calculé avec l'outil de l'OMS était environ neuf fois plus élevé que celui enregistré. Par conséquent, le Paraguay a: (i) proposé des formations afin d'améliorer le diagnostic et le signalement des cas; (ii) renforcé les systèmes d'information pour le suivi et la notification des cas; et enfin, (iii) fourni des doubles tests rapides VIH/syphilis et des tests rapides de la réagine plasmatique supplémentaires en vue d'accroître les capacités de dépistage de la syphilis. En outre, le ministère de la Santé a élaboré un nouveau plan national destiné à éliminer la transmission de la syphilis de la mère à l'enfant, comprenant une série d'étapes de suivi.

**Leçons tirées** Les procédures des prestataires de soins de santé en matière de notification et de surveillance de la syphilis congénitale pourraient différer de la définition des cas aux niveaux national et international. L'utilisation de l'outil d'évaluation mis au point par l'OMS pour la syphilis congénitale au Paraguay a attiré l'attention sur cette maladie qui représente un problème de santé publique nationale, et souligné l'importance des systèmes de surveillance complets et des données précises. Continuer à employer cet outil peut permettre de mesurer les progrès accomplis dans l'élimination de la transmission de la syphilis de la mère à l'enfant, en contribuant à améliorer la couverture des services et la surveillance nationale.

**Método de la OMS para estimar la sífilis congénita con el fin de informar sobre la vigilancia y la prestación de servicios en Paraguay**

**Resumen**

**Situación** En Paraguay, los datos de vigilancia incompletos ocasionaron que se subestimara la carga de sífilis congénita, lo que, a su vez, hizo que se perdieran las oportunidades de diagnóstico y tratamiento de los lactantes.

**Enfoque** La tasa de incidencia de la sífilis congénita, según la definición de la Organización Mundial de la Salud (OMS), se estimó para Paraguay utilizando la herramienta de estimación de la sífilis congénita de la OMS. Esta herramienta también se utilizó para monitorear el progreso hacia la eliminación de la transmisión maternofilial de la sífilis.

**Marco regional** La carga de sífilis en Paraguay ha sido históricamente alta: su prevalencia en embarazadas se estimó en un 3 % en 2018.

**Cambios importantes** La tasa de incidencia de la sífilis congénita estimada con la herramienta de la OMS era aproximadamente nueve veces superior a la tasa de incidencia notificada. En consecuencia, Paraguay: i) impartió formación para mejorar el diagnóstico y la notificación de los casos; ii) reforzó los sistemas de información para el monitoreo y la notificación de los casos; y iii) adquirió más pruebas rápidas duales de VIH-sífilis y de inmunoglobulina E plasmática rápida para aumentar la capacidad de realizar pruebas de sífilis. Además, el Ministerio de Salud Pública preparó un nuevo plan nacional para eliminar la transmisión maternofilial de la sífilis, con claros hitos de monitoreo.

**Lecciones aprendidas** Es posible que los procedimientos de notificación y vigilancia de la sífilis congénita que aplican los profesionales sanitarios no reflejen de manera adecuada las definiciones nacionales e internacionales de los casos. El uso de la herramienta de estimación de la sífilis congénita de la OMS en Paraguay llamó la atención sobre la sífilis congénita como un problema nacional de salud pública y destacó la importancia de contar con sistemas nacionales de vigilancia integrales y datos precisos. El uso constante de la herramienta de la OMS puede hacer un seguimiento de los avances hacia la eliminación de la transmisión maternofilial de la sífilis al ayudar a mejorar la cobertura de los servicios relacionados con la sífilis y la vigilancia nacional.

**طريقة منظمة الصحة العالمية لتقييم الزهري الخلقي بغرض المراقبة وتقديم الخدمات، باراغواي**

**ملخص**

**المشكلة** أدت بيانات المراقبة غير المكتملة في باراغواي إلى الحد من عبء تقييم الزهري الخلقي، مما أدى بدوره إلى ضياع فرص تشخيص الرضع وعلاجهم.

**الأسلوب** تم تقييم الإصابة بمرض الزهري الخلقي، وفقًا لتعريف منظمة الصحة العالمية (WHO)، في باراغواي باستخدام أداة تقييم الزهري الخلقي التابعة لمنظمة الصحة العالمية. تم استخدام هذه الأداة أيضًا لرصد التقدم نحو القضاء على انتقال مرض الزهري من الأم إلى الطفل.

**المواقع المحلية** كان عبء مرض الزهري في باراغواي مرتفعًا على مدى تاريخها، فقد تم تقدير انتشاره بين النساء الحوامل بنسبة 3% في عام 2018.

**التغيّرات** **ذات** **الصلة** كان معدل الإصابة بمرض الزهري الخلقي، الذي تم تقييمه باستخدام أداة منظمة الصحة العالمية، حوالي تسعة أضعاف الإصابة المُبلغ عنها. وبالتالي فقد قامت باراغواي بما يلي: (1) توفير التدريب لتحسين التشخيص والإبلاغ عن الحالات؛ و(2) تعزيز نظم المعلومات لرصد الحالات والإبلاغ عنها؛ و(3) شراء اختبارات إضافية سريعة مزدوجة لفيروس نقص المناعة البشرية والزهري، واختبارات البلازما السريعة لزيادة القدرة على اختبار الزهري. وبالإضافة إلى ذلك، فقد أعدت وزارة الصحة خطة وطنية جديدة للقضاء على انتقال مرض الزهري من الأم إلى الطفل، مع مراحل مراقبة واضحة.

**الدروس** **المستفادة** قد لا تعكس إجراءات الإبلاغ والمراقبة لدى مقدمي الرعاية الصحية، والخاصة بمرض الزهري الخلقي، تعريفات الحالة الوطنية والدولية بشكل كافٍ. إن استخدام أداة تقييم الزهري الخلقي التابعة لمنظمة الصحة العالمية في باراغواي، قد لفت الانتباه إلى الزهري الخلقي باعتباره مشكلة وطنية للصحة العامة، وأكّد على أهمية أنظمة المراقبة الوطنية الشاملة والبيانات الدقيقة. إن الاستخدام المستمر لأداة منظمة الصحة العالمية يمكنه أن يتتبع التقدم نحو القضاء على انتقال مرض الزهري من الأم إلى الطفل، وذلك من خلال المساعدة في تحسين تغطية خدمة مرض الزهري والمراقبة الوطنية.

**世界卫生组织先天性梅毒评估方法为巴拉圭的疾病监测和服务供给提供依据**

**摘要**

**问题** 在巴拉圭,不完整的监测数据导致先天性梅毒的负担被低估,由此导致错过婴儿诊断和治疗的机会。

**方法** 根据世界卫生组织的 (WHO) 定义,采用世界卫生组织先天性梅毒评估工具来估算先天性梅毒的发病率。该工具还可用来监测梅毒母婴传播评估领域的进展情况。

**当地状况** 巴拉圭的梅毒负担历来很高:2018 年的孕妇患病率估计占到 3%。

**相关变化** 使用世界卫生组织工具评估的先天性梅毒发病率大约是所报告的发病率的九倍。随后,巴拉圭:(I) 开展了培训以提升诊断和病例报告;(ii) 加强了病例监测和报告的信息系统,并且 (iii) 采购了额外的快速双重 HIV-梅毒检测和快速血浆反应素试验以增强梅毒检测能力。除此以外,卫生部还制定了一项新的全国性计划,通过明确的监测里程碑来评估梅毒的母婴传播。

**经验教训**医疗护理机构的先天性梅毒报告和监测程序可能无法充分反映全国性和国际性的病例定义。在巴拉圭采用世界卫生组织先天性梅毒评估工具提高了对先天性梅毒作为全国性公共卫生问题的关注度,强调了综合的全国性监测系统和准确数据的重要性。持续使用世界卫生组织工具可以有助于提高梅毒卫生服务覆盖面和全国性监测的普及,从而追赶梅毒母婴传播评估领域的进展。

**Метод ВОЗ для оценки врожденного сифилиса с целью наблюдения и предоставления услуг, Парагвай**

**Резюме**

**Проблема** В Парагвае неполные данные наблюдений привели к недооценке бремени врожденного сифилиса, что в свою очередь привело к упущенным возможностям в диагностике и лечении младенцев.

**Подход** В Парагвае частота встречаемости врожденного сифилиса, по определению Всемирной организации здравоохранения (ВОЗ), оценивалась с использованием инструмента ВОЗ для оценки врожденного сифилиса. Этот инструмент также использовали для мониторинга прогресса в борьбе с передачей сифилиса от матери к ребенку.

**Местные условия** В Парагвае бремя сифилиса достигло рекордных показателей: его распространенность среди беременных женщин в 2018 году составила 3%.

**Осуществленные перемены** Частота встречаемости врожденного сифилиса, оцененная с помощью инструмента ВОЗ, примерно в девять раз превышала зарегистрированную частоту встречаемости. В результате Парагвай: (i) организовал обучение для улучшения диагностики и отчетности о случаях заболевания; (ii) укрепил информационные системы для мониторинга случаев заболевания и отчетности; (iii) закупил дополнительные двойные экспресс-тесты на ВИЧ/сифилис и реакцию быстрого определения реагинов плазмы, чтобы расширить возможности тестирования на сифилис. Кроме того, Министерство здравоохранения подготовило новый национальный план по борьбе с передачей сифилиса от матери к ребенку с четкими этапами мониторинга.

**Выводы** Процедуры отчетности и наблюдения медицинских работников за врожденным сифилисом могут неадекватно отражать определения случаев заболевания на национальном и международном уровне. Использование инструмента ВОЗ для оценки врожденного сифилиса в Парагвае привлекло внимание к врожденному сифилису как национальной проблеме в сфере общественного здравоохранения и подчеркнуло важность комплексных национальных систем наблюдения и точных данных. Постоянное использование инструмента ВОЗ поможет отслеживать прогресс в борьбе с передачей сифилиса от матери к ребенку, способствуя большему охвату услугами по борьбе с сифилисом и наблюдению на национальном уровне.

